# Geographical distribution of ALDH2 rs671 polymorphism in Chinese angina pectoris patients

**DOI:** 10.3389/fgene.2025.1543963

**Published:** 2025-09-05

**Authors:** Wenjia Dong, Junjie Yu, Siqi Xu, Hongsheng Li

**Affiliations:** Department of Clinical Laboratory, The Second Hospital of Jiaxing, Zhejiang, China

**Keywords:** ALDH2 polymorphism, angina pectoris, regional variation, clinical characteristics, nitroglycerin

## Abstract

**Background:**

Mitochondrial acetaldehyde dehydrogenase 2 (ALDH2) plays a critical role in the metabolism of ethanol and nitroglycerin. Mutations in ALDH2 reduce enzymatic activity, impairing nitroglycerin metabolism. ALDH2 genetic polymorphisms exhibit significant regional variations across China. This study investigates the distribution of ALDH2 allelic and genotypic frequencies in patients with angina pectoris across northern (Luoyang, Henan), eastern (Jiaxing, Zhejiang), and southern (Guilin, Guangxi) China, and explores the relationship between ALDH2 genotypes and selected clinical characteristics.

**Materials and Methods:**

A total of 1,084 angina pectoris patients were recruited from the three aforementioned regions. ALDH2 genotyping was performed using fluorescence quantitative PCR, and associations between genotypes and clinical characteristics, such as smoking and alcohol consumption histories, were analyzed.

**Results:**

The geographical distribution of the ALDH2 rs671 G→A mutation showed a significant increase in mutation frequency from northern to southern China. Genotypic distributions differed significantly across regions, with GG being predominant in northern China and higher frequencies of GA and AA observed in southern regions. Additionally, lifestyle factors such as smoking and alcohol consumption were significantly associated with the presence of the A allele, reflecting the interplay between genetics and environmental influences.

**Conclusion:**

This study reveals significant regional differences in ALDH2 rs671 polymorphism and its association with lifestyle factors in angina pectoris patients across China. Since the ALDH2*2 variant markedly reduces the metabolic activation of nitroglycerin, these findings have important clinical implications: in regions with a higher prevalence of the A allele, patients may exhibit reduced therapeutic response to nitroglycerin. This highlights the need for region-specific and genotype-informed strategies in the management of angina pectoris.

## Introduction

ALDH2 is localized within the mitochondria, which are situated on chromosome 12 (12q24.4). It consists of 13 exons and 12 introns, encoding a protein of 500 amino acids (Li et al., 2006). ALDH2 exhibits both aldehyde dehydrogenase and esterase activities, playing a pivotal role as a key enzyme in the metabolic pathways of ethanol and nitroglycerin drugs (Luo, 2022; Cai et al., 2023). Moreover, it possesses cardioprotective properties by reducing myocardial cell apoptosis (Pan et al., 2021; Mizuno et al., 2016a; Xia et al., 2020). Studies investigating the genetic polymorphism of ALDH2 have elucidated that the predominant genotype at locus ra671 is GG (wild-type ALDH2). However, when carrying the mutant allele ALDH2*2 polymorphism, there occurs a substitution from glutamic acid (Glu) to lysine (Lys) at position 504 (rs671, ALDH2*2 or Glu504Lys) (Crabb et al., 1989; Zhao, 2019; Yoshida et al., 1995). This mutation impairs the cofactor binding ability of ALDH2, leading to decreased enzymatic activity. Heterozygous individuals retain only about 10% of the enzyme activity compared to wild-type individuals, while homozygous mutants completely lack enzymatic activity (Lai et al., 2014).

The mutation of ALDH2 results in a reduction in enzyme activity, leading to impaired alcohol breakdown and the development of alcohol metabolism-related diseases such as liver damage (Bosron et al., 1988) and various types of cancer (Zhang and Fu, 2021; Wang et al., 2020). Moreover, polymorphisms in the ALDH2 gene are associated with the occurrence and progression of coronary artery disease (Tang, 2024). The ALDH2 variant reduces enzymatic activation of nitroglycerin, a key drug used for angina symptom relief, leading to decreased vasodilatory effects. For instance, Zhang et al. demonstrated that patients with mutant ALDH2 alleles had significantly lower changes in coronary artery diameter and systolic blood pressure after nitroglycerin administration compared to wild-type individuals (Zhang, 2025). Moreover, the East Asian-specific ALDH22 genotype has been repeatedly associated with increased risk of coronary spastic angina, a subtype of angina pectoris, and diminished therapeutic response to nitrate-based therapy (Mizuno et al., 2015). Additionally, among patients with coronary spastic angina, those with the ALDH22 genotype had markedly reduced nitroglycerin-mediated dilation and increased nitrate tolerance following continuous glyceryl trinitrate administration, compared to ALDH21 homozygote (Mizuno et al., 2020). Finally, Miura et al. conducted a randomized crossover trial in Japanese volunteers with different ALDH2 genotypes and found that individuals carrying the mutant ALDH2*2 allele exhibited approximately 40% less vasodilatory response to nitroglycerin compared to wild-type controls, reinforcing the genotype-dependent variation in nitrate efficacy (Xia et al., 2015).

These pharmacogenetic findings underscore the importance of considering ALDH2 genotype when prescribing nitrate therapy for angina pectoris. Given the clear clinical consequences of the ALDH2*2 allele on nitroglycerin metabolism and response (Min and Kitakaze, 2020), it becomes crucial to investigate not only its presence but also its regional distribution among patient populations. Understanding such geographical patterns may help optimize individualized treatment strategies across different regions of China. Previous studies have identified significant ethnic and geographic differences in the distribution of ALDH2 gene polymorphisms (Cook, 2021; Tokiya, 2023; Chen et al., 2022; Li et al., 2009). However, the geographical distribution of ALDH2 polymorphisms in Chinese patients with cardiovascular diseases remains largely unexplored.

This study aims to fill this gap by examining the genotypic and allelic frequencies of ALDH2 in angina pectoris patients from three geographically distinct regions—northern, eastern, and southern China. Our findings reveal a clear geographical trend, with a higher frequency of the G→A mutation observed in the southern regions compared to the north. These regional variations have important clinical implications, as they may affect the metabolism of nitroglycerin and other drugs that rely on ALDH2 activity, making it essential to consider these genetic differences when prescribing treatment.

In addition to examining the geographical distribution of ALDH2 polymorphisms, it is essential to explore how these genetic variations interact with clinical and lifestyle factors in angina pectoris patients. Previous studies have highlighted the potential influence of lifestyle behaviors, such as smoking and alcohol consumption, on the prevalence of ALDH2 mutations (Chen et al., 2022; Ma et al., 2017). To better understand these interactions, this study also analyzed the relationship between ALDH2 genotypes and selected clinical characteristics, including patient histories of smoking, alcohol consumption, and hypertension, as well as laboratory indicators. These findings provide valuable insights into the interplay between genetic predisposition and environmental factors, which could have significant implications for personalized treatment strategies in angina pectoris patients.

## Materials and methods

### Study participants

Patients diagnosed with angina pectoris and admitted to the First Affiliated Hospital of Henan University of Science and Technology, the Second Affiliated Hospital of Jiaxing University, and Guilin Medical College Second Affiliated Hospital between 1 September 2021, and 31 August 2023 were included in this study. We included all clinically classified angina pectoris patients, including those with stable, unstable, and variant angina pectoris, without distinguishing between specific subtypes or imposing comorbidity restrictions. There were no restrictions based on gender, age, or ethnicity. A total of 1,084 patients residing in local counties or cities were screened; among them were 359 from Luoyang, 352 from Jiaxing, and 373 from Guilin. The cohort consisted of 650 male patients and 434 female patients overall. Specifically, for Luoyang, there were 214 males and 145 females with an average age of 65.05 years; for Jiaxing, there were 189 males and 163 females with an average age of 64.94 years; for Guilin there were 247 males and 126 females with an average age of 67.51 years. Although the ages ranged from 29 to 97 years old among angina pectoris patients, the majority belonged to middle-aged and elderly individuals.

### Definition of variables

The primary outcome of this study was the distribution of ALDH2 rs671 genotypes (GG, GA, AA) across different geographic regions and their associations with selected clinical characteristics. Exposure variables included geographic region (northern, eastern, and southern China) and patient lifestyle factors, such as smoking and alcohol consumption. Clinical predictors analyzed in relation to ALDH2 genotypes included history of hypertension, diabetes, and several laboratory parameters. Potential confounders, including age and gender, were recorded but not adjusted for in the statistical analysis. The diagnosis of angina pectoris was based on clinical symptoms, electrocardiogram (ECG) findings, and/or coronary angiography results, according to standard cardiology guidelines at the respective institutions.

### Detection of ALDH2 gene polymorphism

Peripheral blood samples from angina pectoris patients were promptly processed for ALDH2 gene polymorphism detection upon collection. Genomic DNA was extracted from whole blood using a commercially available kit (Beijing Tiangen Biochemical Technology Co., Ltd., Shanghai, China). The ALDH2 rs671 gene polymorphism was determined using a human-specific detection kit (Wuhan Kanglu Biotechnology Co., Ltd., Wuhan, China) based on the PCR-fluorescent probe method, following the manufacturer’s instructions.

### Detection of clinical observation indicators

During hospitalization, comprehensive clinical information of patients with ALDH2 gene polymorphism was collected, including gender, smoking history, drinking history, hypertension history, diabetes history, as well as laboratory indicators such as hypersensitive C-reactive protein (hsCRP), total bilirubin (TBIL), direct bilirubin (DBIL), alkaline phosphatase (ALT), triglyceride (TG), lactate dehydrogenase (LDH), and homocysteine (HCY). Furthermore, the study compared the differences in laboratory indicators between patients with angina pectoris and other patients in the cardiovascular department or undergoing routine physical examinations.

### Bias control

To minimize potential sources of bias, we used the same standardized PCR-fluorescent probe method for ALDH2 genotyping across all three centers. Clinical and laboratory data were extracted from hospital electronic medical records using predefined criteria, reducing information bias. Patients were consecutively enrolled during the study period to mitigate selection bias. Furthermore, all three hospitals followed national laboratory protocols, ensuring consistency and comparability of biochemical measurements. Hardy-Weinberg equilibrium analysis was conducted to confirm the representativeness of the genotypic distribution within each regional population.

### Statistical analysis

Data were analyzed using SPSS 25.0 (IBM, Armonk, NY, United States). The Shapiro-Wilk test was used to assess the normality of quantitative variables. Normally distributed data were expressed as mean ± standard deviation and analyzed using Student’s t-test or one-way ANOVA. Non-normally distributed data were analyzed using the Wilcoxon rank-sum test or Kruskal–Wallis test. Categorical variables were analyzed using the Chi-square test. A significance level of P < 0.05 was considered statistically significant.

No multivariable regression or confounder-adjusted analyses were conducted. No subgroup or interaction analyses were performed. Complete case analysis was used, and no missing data imputation was necessary. Patients were enrolled consecutively without sampling randomization. No sensitivity analyses were performed in this study.

## Results

### Hardy-Weinberg equilibrium analysis of ALDH2 genotypes in angina pectoris patients across three Chinese regions

A total of 1,084 patients diagnosed with angina pectoris were consecutively recruited from three hospitals between September 2021 and August 2023. All patients who met the inclusion criteria were included in the final analysis. There were no dropouts or exclusions during the study period. Therefore, no formal flow diagram was used.

**FIGURE 1:**
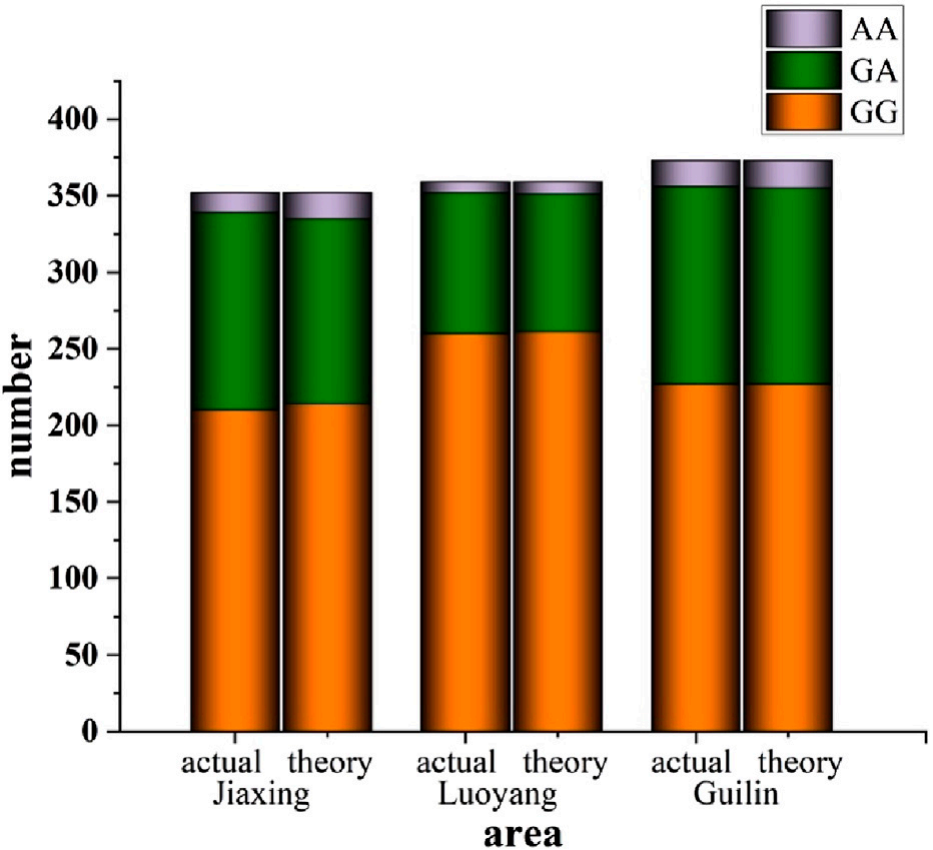
Equilibrium test of ALDH2 gene polymorphisms.

The ALDH2 loci data from populations in Jiaxing, Luoyang, and Guilin were subjected to Hardy-Weinberg equilibrium analysis. For instance, in the Jiaxing region, the observed genotype counts were 210 for GG, 129 for GA, and 13 for AA. Consequently, the observed genotype frequencies were 59.66% for GG, 36.65% for GA, and 3.69% for AA, with corresponding allele frequencies of 77.99% for G and 22.01% for A. According to Hardy-Weinberg equilibrium expectations, the theoretical genotype counts would be 214 for GG, 121 for GA, and 17 for AA. A comparison between the observed and expected genotype counts is presented in Table 1 and illustrated in Figure 1. The χ2 value of 0.827 and P-value of 0.661 (*P* > 0.05) suggest that the sample from Jiaxing adheres to the Hardy-Weinberg equilibrium, indicating that it is representative of the population in this region. Similar findings were observed in the Luoyang (*P* = 0.956) and Guilin (*P* = 0.984) regions, where both P-values also exceeded the significance level of α = 0.1, further supporting the representativeness of these samples.

**TABLE 1:** Equilibrium test of ALDH2 gene polymorphisms.

Area	Number	Genotypes			c^2^	*P*
		GG	GA	AA		
Jiaxing	Observed	210	129	13	0.827	0.661
	Theory	214	121	17		
Luoyang	Observed	260	92	7	0.091	0.956
	Theory	261	90	8		
Guilin	Observed	227	129	17	0.032	0.984
	Theory	227	128	18		

### Regional variations in ALDH2 polymorphism among angina pectoris patients in China

**FIGURE 2:**
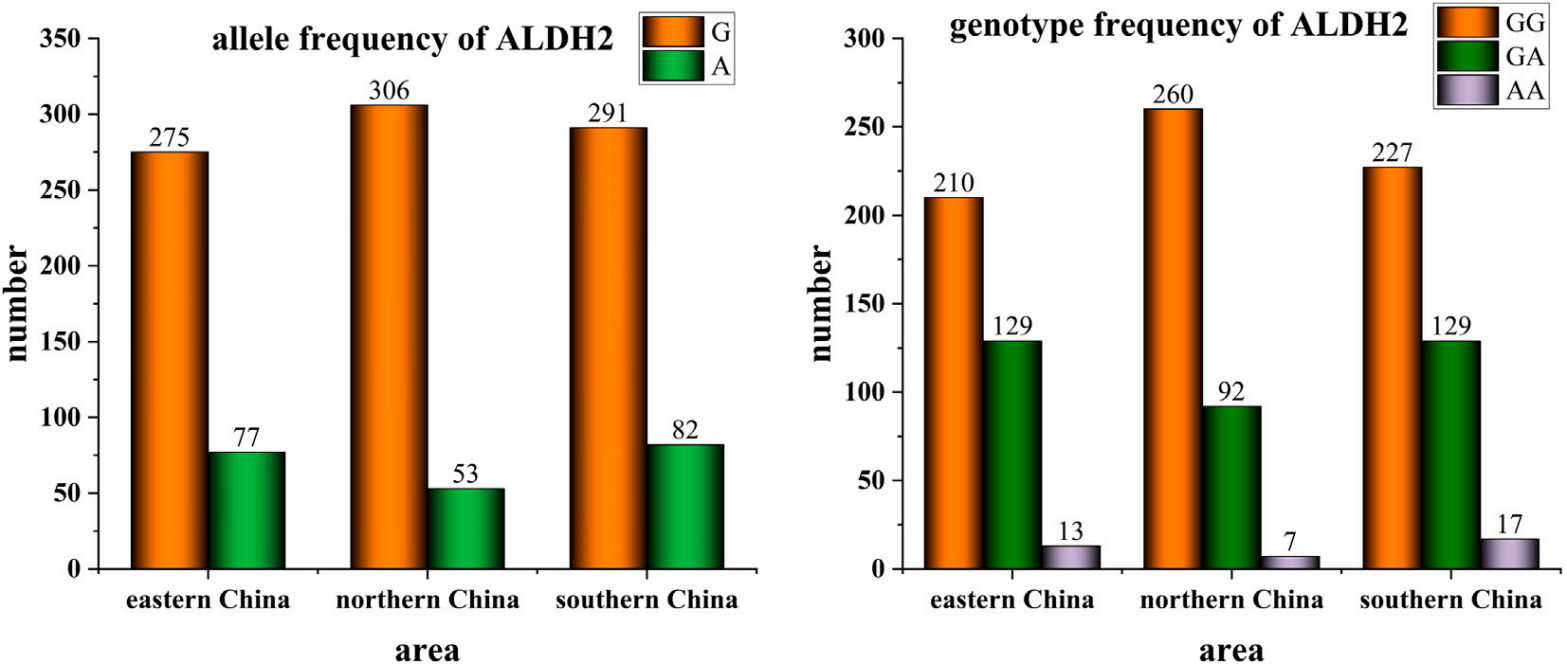
Allele and genotype frequency of ALDH2 in individuals with angina pectoris from different geographical regions of China.

This study detected the ALDH2 gene polymorphism in 352 angina pectoris patients treated at the Second Hospital of Jiaxing, 359 angina pectoris patients treated at the First Affiliated Hospital of Henan University of Science and Technology, and 373 angina pectoris patients treated at the Second Affiliated Hospital of Guilin Medical University from September 2021 to August 2023. In eastern China (Jiaxing), the G and A allele frequencies were 77.99% and 22.01%, respectively, with the wild-type GG genotype occurring in 59.66% of cases, the heterozygous GA genotype in 36.65%, and the homozygous AA genotype in 3.69%. In northern China (Luoyang), the G and A allele frequencies were 85.25% and 14.75%, respectively, with GG, GA, and AA genotype frequencies of 72.47%, 25.56%, and 1.97%. In southern China (Guilin), the G and A allele frequencies were 78.09% and 21.91%, respectively, with corresponding GG, GA, and AA genotype frequencies of 60.75%, 34.68%, and 4.57% (Table 2). Figure 2 provides a visual representation of these disparities.

**TABLE 2:** Allele and genotype frequency of ALDH2 [n (%)].

Area	n	Allele		Genotype		
		G	A	GG	GA	AA
Eastern China	352	275 (77.99%)	77 (22.01%)	210 (59.66%)	129 (36.65%)	13 (3.69%)
Northern China	359	306 (85.25%)	53 (14.75%)	260 (72.47%)	92 (25.56%)	7 (1.97%)
Southern China	373	291 (78.09%)	82 (21.91%)	227 (60.75%)	129 (34.68%)	17 (4.57%)

Comparing the detection results from the three regions (Table 3), statistically significant differences were observed in the ALDH2 genotypes among populations in northern, eastern, and southern China. Consequently, a gradual increasing trend was observed in the proportion of G→A mutations from north to south. The G→A mutation at the ALDH2 rs671 locus leads to a reduction in ALDH2 activity, with heterozygous individuals exhibiting only approximately 10% of the enzyme activity seen in wild-type individuals, while homozygous mutant individuals completely lack enzymatic activity. Therefore, individuals carrying the A allele have diminished capacity for alcohol metabolism and experience discomfort such as flushing and accelerated heartbeat even with minimal alcohol consumption. The geographical variations in gene polymorphism at the ALDH2 rs671 locus align with current observations that people residing in northern China tend to exhibit greater tolerance towards alcohol compared to those living in southern regions.

**TABLE 3:** Genotype frequency of ALDH2 by regions [n (%)].

Area	n	Genotype			χ^2^	P
		GG	GA	AA		
Northern China	359	260 (72.47%)	92 (25.56%)	7 (1.97%)		
Eastern China	352	210 (59.66%)	129 (36.65%)	13 (3.69%)	13.25	0.001
Northern China	359	260 (72.47%)	92 (25.56%)	7 (1.97%)		
Southern China	373	227 (60.75%)	129 (34.68%)	17 (4.57%)	12.33	0.002
Eastern China	352	210 (59.66%)	129 (36.65%)	13 (3.69%)		
Southern China	373	227 (60.75%)	129 (34.68%)	17 (4.57%)	0.587	0.746

### The relationship between ALDH2 genotype and clinical parameters

The geographical distribution of ALDH2 polymorphisms among angina pectoris patients in three regions of China has been the focus of our research, While these variations offer valuable insights into regional genetic disparities, understanding how these polymorphisms relate to specific clinical characteristics is crucial for assessing their broader implications. Identifying these associations can shed light on the role of ALDH2 genotypes in influencing lifestyle-related risk factors, metabolic profiles, and disease progression, ultimately guiding more targeted and effective treatment strategies.

To further explore the relationship between ALDH2 genotypes and clinical characteristics, we conducted a comparative analysis of various factors among ALDH2-mutated patients in East China. These factors included gender, smoking history, alcohol consumption, hypertension, diabetes, and laboratory indicators such as hsCRP, total TBIL, DBIL, ALT, TG, LDH, and HCY. As summarized in Table 4, individuals with a smoking habit, alcohol consumption, or a history of hypertension were more likely to carry genetic mutations, and these differences were statistically significant. However, no significant associations were observed between gender or laboratory indicators and genotype. This lack of significance may be attributed to the higher proportion of male participants in our sample, as well as notable variations in participant distribution across the different genotypes.

**TABLE 4:** Comparison of clinical characteristics across ALDH2 genotypes in East China.

Variable	GG	AG	AA	χ^2^	P
Number	210	129	13		
Gender, male (%)	117 (55.9%)	68 (52.7)	4 (30.8%)	3.219	0.200
Current smoking, n (%)	82 (39%)	44 (34.1%)	1 (7.7%)	6.056	0.048
Drinking, n (%)	71 (33.8%)	20 (15.5%)	2 (15.4%)	14.620	0.001
Hypertension, n (%)	112 (44.8%)	78 (60.5%)	5 (38.5%)	9.038	0.011
Diabetes, n (%)	58 (27.6%)	43 (33.3%)	1 (7.7%)	4.239	0.120
hsCRP (mg L^-1^)	4.18 ± 8.51	5.57 ± 13.00	7.66 ± 13.26	1.325	0.515
TBIL (μmol L^-1^)	13.95 ± 6.40	12.67 ± 5.59	11.65 ± 4.08	4.401	0.111
DBIL (μmol L^-1^)	4.74 ± 2.32	4.71 ± 2.16	4.48 ± 1.86	0.029	0.986
ALP (U L^-1^)	76.23 ± 21.97	78.50 ± 34.45	74.08 ± 17.53	0.027	0.987
TG (mmol L^-1^)	1.71 ± 1.80	1.62 ± 0.97	1.15 ± 0.39	4.357	0.113
LDH (U L^-1^)	187.10 ± 43.21	193.62 ± 74.62	179.62 ± 26.30	1.709	0.426
HCY (μmol L^-1^)	12.68 ± 6.42	12.99 ± 6.34	10.57 ± 3.68	1.512	0.470

## Discussion

ALDH2 plays a crucial role in the bioactivation of nitroglycerin by catalyzing its conversion to nitric oxide (NO), a potent vasodilator that alleviates myocardial ischemia. This mitochondrial dehydrogenase reduces organic nitrates to NO, which activates soluble guanylate cyclase in vascular smooth muscle (Opelt et al., 2016). However, the ALDH2 rs671 polymorphism (Glu504Lys) markedly impairs this enzymatic activity—heterozygotes retain only ∼10% of wild-type function, while homozygous mutants lose nearly all activity—leading to reduced nitroglycerin bioactivation and diminished vasodilatory response. Moreover, a 2022 study showed that continuous nitroglycerin use in ALDH2 variant carriers exacerbates nitrate tolerance and vascular dysregulation (Maiuolo, 2022), highlighting the clinical implications of this pharmacogenetic interaction.

Previous studies have established that there are significant regional differences in the distribution of the ALDH2 G-to-A mutation between northern and southern China among the general population (Zhang et al., 2021). Pecifically, a higher proportion of individuals in southern China carry the A allele, leading to reduced ALDH2 enzyme activity. This geographical variation has been linked to differences in alcohol tolerance across these regions, with northern populations exhibiting greater tolerance (Cao, 2020). Despite this known variation in the general population, the distribution of ALDH2 genotypes among patients with angina pectoris had not been thoroughly investigated before this study. This gap in research is particularly important because ALDH2 plays a crucial role in the metabolism of nitroglycerin, a key medication used to relieve angina symptoms. The G-to-A mutation at the ALDH2 rs671 locus results in reduced or absent enzyme activity, significantly impairing the conversion of nitroglycerin into nitric oxide, which is necessary for vasodilation and pain relief in angina patients.

Our study found that the distribution of the ALDH2 G-to-A mutation in patients with coronary heart disease varies significantly between northern and southern China, as well as between eastern and northern China. Specifically, the chi-square test revealed significant differences between eastern and northern regions (*P* = 0.001) and between northern and southern regions (P = 0.002), but no significant difference between eastern and southern regions. These findings suggest that the previously observed geographical variations in the general population extend to patients with coronary heart disease, potentially affecting the efficacy of nitroglycerin treatment across different regions.

Our study also revealed significant associations between ALDH2 genotypes and lifestyle factors, as shown in Table 4. Individuals with a history of smoking, alcohol consumption, or hypertension were more likely to carry the A allele, highlighting the influence of environmental factors on the prevalence of ALDH2 mutations. Notably, previous studies have indicated that the ALDH2 A allele may not only be more prevalent among individuals with certain lifestyle habits, but may also amplify the biological effects of exposures such as smoking and alcohol, further contributing to cardiovascular risk. ALDH2 deficiency leads to accumulation of toxic aldehydes like acetaldehyde and 4-HNE, which are normally cleared by the wild-type enzyme. In individuals who consume alcohol, especially those carrying the A allele, acetaldehyde accumulates and induces oxidative stress, endothelial dysfunction, and sympathetic activation, which can exacerbate cardiac ischemia (Chen et al., 2010; Zhang, 2023). Similarly, smoking generates reactive aldehydes and ROS, and the lack of effective detoxification in ALDH22 carriers may potentiate inflammation and vascular injury (Tsai et al., 2020; Mizuno et al., 2016b). Therefore, beyond pharmacogenetics, the A allele may influence angina risk and disease progression through impaired detoxification and greater vulnerability to environmental toxins. These findings underscore the interplay between genetic and environmental factors in angina pectoris patients and highlight the need for further research to refine treatment strategies based on these interactions. However, the relatively small number of AA genotype carriers (n = 13) limits the statistical power of subgroup comparisons and raises the possibility of false-negative or unstable estimates. Therefore, these associations should be interpreted with caution. Further large-scale, multi-center studies are needed to confirm these preliminary findings and to better understand how genetic and environmental factors jointly influence angina risk and treatment response.

Given these findings, it is essential to consider regional and lifestyle differences in ALDH2 polymorphism when prescribing nitroglycerin for angina pectoris. In areas where the A allele is more prevalent, such as southern China, clinicians may need to monitor patients more closely for nitroglycerin efficacy and explore alternative treatments if standard doses prove insufficient. This study underscores the importance of personalized medicine and highlights the need for further research into the impact of ALDH2 polymorphisms on the treatment of cardiovascular diseases.

## Limitations

This study has several limitations. First, the cohort was restricted to three regions in China—Jiaxing, Luoyang, and Guilin—which may not capture the full genetic diversity of the broader Chinese or global populations. Second, although the overall sample size was reasonable, subgroup analyses (particularly for the AA genotype) were limited by small numbers, which may affect the reliability of certain associations. Third, we did not adjust for potential confounders such as age and gender, nor did we apply corrections for multiple statistical comparisons, which increases the risk of false-positive findings. Lastly, the cross-sectional design limits causal interpretation, and future longitudinal studies are needed to validate these findings and assess their clinical implications.

## Data Availability

The aggregate data (Geographical Distribution of ALDH2 rs671 Polymorphism in Chinese angina pectoris Patients), supporting the findings of this study, are available within this article. The original individual-level genotype datasets generated during and/or analyzed during the current study are not publicly available at this time due to patient privacy and ethical considerations, but are available upon reasonable request from the corresponding author (Siqi Xu, xusiqi0967@zjxu.edu.cn).
